# The Outcomes of Revision Anterior Cervical Decompression and Fusion Using a Stand-Alone Implant Versus Traditional Interbody Polyetheretherketone Cage, Titanium Plate, and Screw Instrumentation

**DOI:** 10.7759/cureus.49246

**Published:** 2023-11-22

**Authors:** Abduljabbar Alhammoud, Paul D Korytkowski, William F Lavelle, Richard A Tallarico

**Affiliations:** 1 Orthopedic Spine Surgery, University of Arizona, Tucson, USA; 2 Orthopedic Surgery, State University of New York Upstate Medical University, Syracuse, USA

**Keywords:** dysphagia, interbody cage, stand-alone implant, revision, adjacent segment disease (asd), anterior cervical decompression and fusion (acdf)

## Abstract

Introduction: Anterior cervical decompression and fusion (ACDF) is the standard surgical procedure for cervical radiculopathy and myelopathy, although ACDF includes risks of adjacent segment disease (ASD) and subsequent revision procedures. Various interbody cage, plate, and screw options can be utilized. Stand-alone devices were designed to overcome undesired complications of hardware prominence and associated dysphagia, soft tissue violation, and adjacent level encroachment. Implants include biomechanical structural support (cage) composed of various materials (polyetheretherketone (PEEK)/titanium) and integral fixation (screws/blades). The purpose was to compare intraoperative, short- and long-term outcomes of revision ACDF using a stand-alone implant (ACDF-ZP group) versus traditional interbody PEEK cage, titanium plate, and screw instrumentation (ACDF-CP group).

Methods: This was a retrospective, cohort study reviewing charts of patients who underwent revision ACDF. The primary outcome measure was the incidence of postoperative dysphagia. Secondary outcomes included intraoperative, short-term, and long-term outcomes and complications.

Results: Sixty-one patients were included (ACDF-ZP group = 50; ACDF-CP group = 11). In-hospital incidence of dysphagia was significantly less in the ACDF-CP group (P = 0.041). Thrity-one (62.0%) of the ACDF-ZP group reported dysphagia postoperatively, half resolved by 6 weeks, and two persisted for more than 6 months. Five (45.5%) of the ACDF-CP group reported dysphagia with most resolving within 6 weeks. There were no statistically significant differences between groups in short- or long-term complications, dysphonia, or reoperation rates. No statistical significance was seen in blood loss, operative time, hospital stay, local and global alignment, or cage subsidence.

Conclusion: Rates of dysphagia were comparable between groups at short and long-term follow-up, despite a greater incidence of postoperative dysphagia in the ACDF-ZP group. All complications and occurrences of cage subsidence were observed in the ACDF-ZP group, which may be attributed to the larger sample size. Given these findings, zero-profile stand-alone implants and traditional interbody PEEK cage, titanium plate, and screw instrumentation appear to be both safe and effective options for revision ACDF.

## Introduction

Anterior cervical decompression and fusion (ACDF) is the standard surgical procedure for cervical radiculopathy and myelopathy. The procedure can be performed utilizing different interbody cage, plate, and screw options. Various interbody biomechanical devices exist, each with distinct advantages and disadvantages [1-3]. Stand-alone devices were first designed to overcome the undesired complication of hardware prominence and its association with dysphagia, soft tissue violation, and adjacent-level encroachment [4-6]. These implants include biomechanical structural support (i.e., cage) composed of various materials (i.e., polyetheretherketone (PEEK)/titanium) and integral fixation (i.e., screws/blades). In a meta-analysis of 10 studies involving 719 cervical spondylosis patients, Dong et al. reported lower rates of complications, early dysphagia, and late dysphagia when ACDF procedures utilized zero-profile implants compared to an anterior plate group, with similar rates of fusion between groups [7].

Adjacent segment disease (ASD) is a well-known complication of spinal surgery. ASD encompasses various complications affecting the spinal level superior or inferior to the fused segment, including listhesis, instability, herniated nucleus pulposus, stenosis, hypertrophic facet arthritis, scoliosis, and vertebral compression fracture [8]. Symptomatic ASD is a significant factor in the decision to perform revision ACDF. In a retrospective cohort study of 672 consecutive patients with a mean follow-up of 31 months, Eck et al. found a revision rate of 15% following ACDF procedures. In their study, revisions were performed for ASD (47.5%), pseudoarthrosis (45.5%), and new problems (7.1%) [9].

Revision ACDF can be accomplished through various techniques, including removal of the plate, extension of the plate, using a separate plate, or using a stand-alone device, sometimes referred to as a zero-profile implant. In a small case series of different techniques for revision ACDF for ASD, Wang et al. found that the low-profile implant was associated with a shorter operative time and less postoperative dysphagia compared to alternative surgical options [10].

Our study aimed to compare the intraoperative, short- and long-term outcomes of revision ACDF utilizing a zero-profile stand-alone implant, the DePuy Synthes ZERO-P stand-alone implant (Raynham, MA, U.S.A, TM: 4038784) versus a traditional interbody PEEK cage, titanium plate, and screw instrumentation.

## Materials and methods

This was a retrospective, comparative cohort study from one academic institution of all revision ACDF procedures utilizing either traditional interbody PEEK cage, titanium plate, and screw instrumentation (ACDF-CP group) or the ZERO-P implant (ACDF-ZP group) from January 2015 until December 2020. The study received an exemption from IRB.

**Figure 1:**
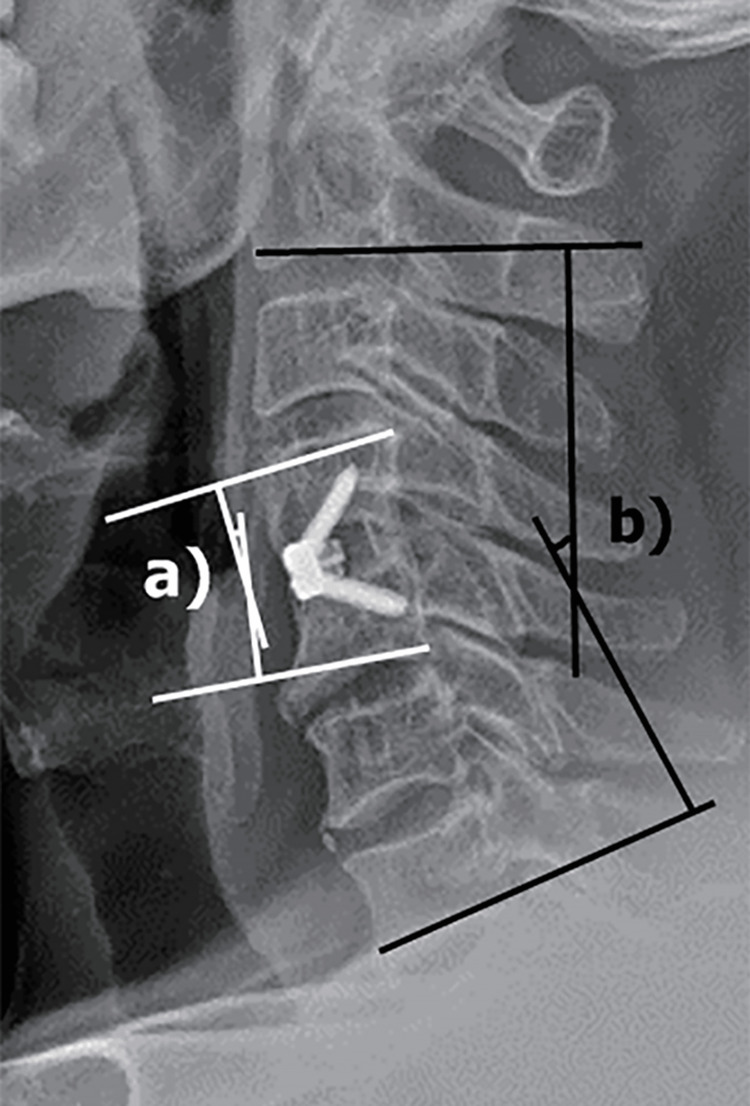
Radiological measurements with (a) depicting local lordosis and (b) depicting global lordosis

The primary outcome was the incidence of dysphagia at different time points; therefore, all patients with at least one follow-up visit were included. Data were collected for demographics (age, gender, indication and level of index procedure, and indications and level of revision procedure), perioperative outcomes (blood loss, operative time, length of hospital stay, complications, and reoperation rate), dysphagia (inpatient incidence, two-weeks, six-weeks, more than six-months postoperatively, and treatment), and radiological parameters (global cervical alignment, local alignment, and cage subsidence). Global alignment was defined as the C2-C7 Cobb angle; local alignment was defined as the Cobb angle at the operated level; and cage subsidence was defined as more than a 2 mm difference in the height at the operated level between postoperative x-ray and final follow-up x-ray (Figure 1).

**Figure 2:**
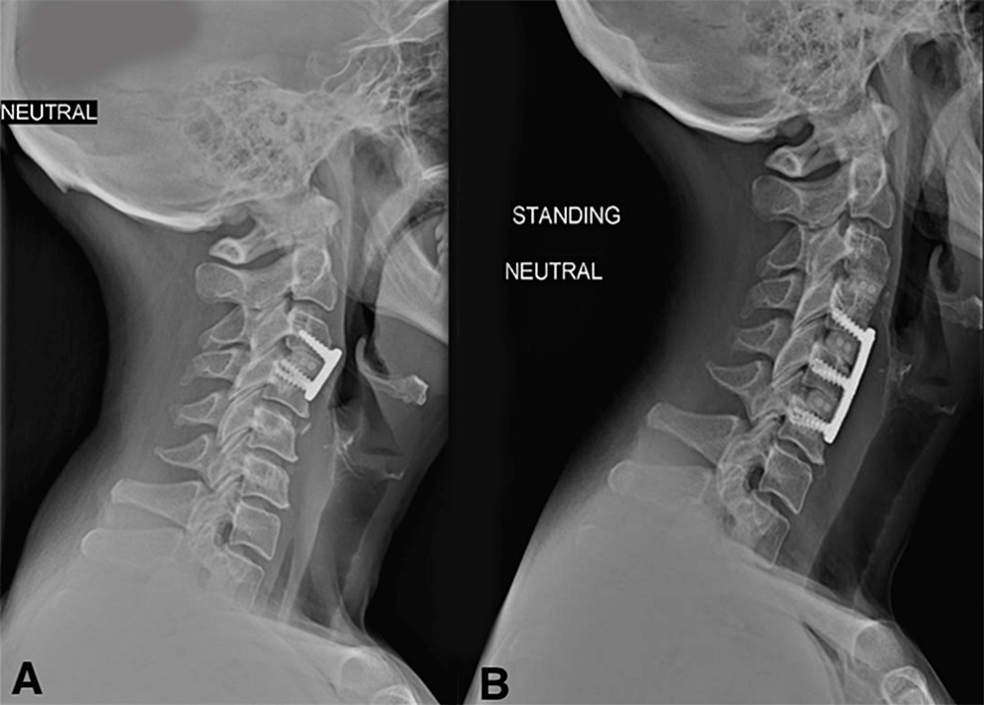
Anteroposterior radiograph of a case presentation of revision ACDF using traditional interbody PEEK cage, titanium plate, and screw instrumentation ACDF, anterior cervical decompression and fusion; PEEK, polyetheretherketone

**Figure 3:**
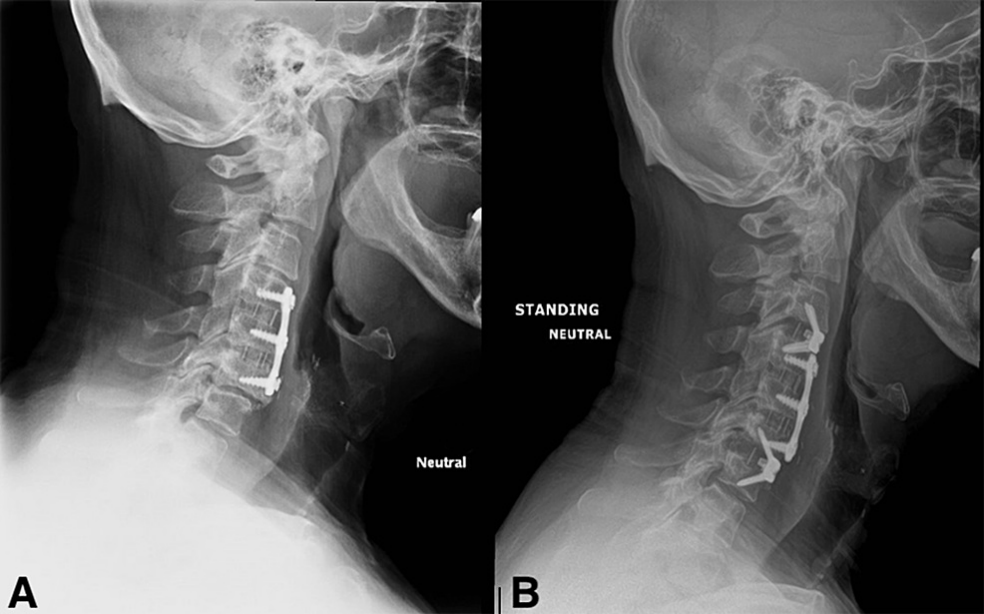
Anteroposterior radiograph of a case presentation of revision ACDF using ZERO-P implant ACDF, anterior cervical decompression and fusion

The surgical technique was a standard revision ACDF approach followed by surgeon preference of implant selection, either traditional interbody PEEK cage, titanium plate, and screw instrumentation after removal of index level plate or a ZERO-P implant without interfering with the index level implant (Figure 2, Figure 3). Patients were not randomized for which implant they received. Two senior surgeons at an academic center performed the revision procedures, one performing all the ZERO-P implant procedures and the other performing all the traditional constructs. Every procedure was performed without assistance from otorhinolaryngology.

Statistical analyses were conducted using statistical packages Statistical Package for Social Sciences (SPSS) version 23.0 (IBM Corp., Armonk, NY) and Epi InfoTM 2000 (Centers for Disease Control and Prevention, Atlanta, GA). Descriptive statistics were used to summarize demographic and radiological measurements. Chi-square test and Fisher’s exact test were used to express the associations between two or more qualitative variables as appropriate. An unpaired t-test was used to compare the quantitative data between the two groups. Frequency (percentage) and mean ± SD or median and range were used as appropriate for categorical and continuous values. A P-value of <0.05 was considered statistically significant.

## Results

Sixty-one patients met the inclusion criteria, with 50 in the ACDF-ZP group (mean age + SD: 60.1 + 11.4 years; male = 25 (50.0%)) and 11 in the ACDF-CP group (mean age + SD: 50.7 + 6.3 years; male = 3 (27.2%)) (Table 1). From the demographical data, overall cervical spondylotic stenosis was the most common indication for the index procedure, followed by cervical myelopathy and cervical radiculopathy. Between the two groups, significantly more ACDF-ZP group patients were diagnosed with cervical spondylosis versus the ACDF-CP group (P = 0.001). Also significant, six (54.5%) ACDF-CP patients compared to three (6.0%) ACDF-ZP patients were diagnosed with cervical radiculopathy, and no ACDF-CP patients had cervical myelopathy versus ten (20.0%) of ACDF-ZP patients. Most of the index and revision procedures were one or two levels with a comparable overall number of levels. However, there was significance in that all ACDF-CP patients only had one level in the index surgery (mean number of levels + SD = 1.0 + 0.0), whereas, the ACDF-ZP group had (mean number of levels + SD = 1.7 + 0.6) levels, P=0.001. In revision surgeries, 35 (70.0%) of the ACDF-ZP group versus one (9.1%) of the ACDF-CP group had one level surgery with significantly more ACDF-CP patients (8; 72.7%) versus ACDF-ZP patients (14; 28.0%) had two level revision surgery, P<0.001. The time from index surgery to revision surgery was significantly shorter in the ACDF-CP group compared to the ACDF-ZP group (P = 0.001). Adjacent segment failure was the most common indication for revision surgery. Approximately 31 (50.8%) of the overall revisions were proximal to the index level, 17 (27.9%) were distal to the index level, and 10 (16.4%) were combined proximal and distal to the index level.

**Table 1:** Comparison of patient demographic data *A P-value of <0.05 was considered statistically significant. SCI, spinal cord injury; PEEK, polyetheretherketone

	N (%)	Zero-profile implant (ACDF-ZP Group)	Traditional PEEK interbody cage, plate, and screws (ACDF-CP Group)	P-value
Number of Patients (%)	61 (100.0%)	50 (82.0%)	11 (18.0%)	
Mean Age ± SD (years)	58.4 ± 11.2	60.1 ± 11.4	50.7 ± 6.3	0.110
Male (%)	28 (45.9%)	25 (50.0%)	3 (27.2%)	0.150
Female (%)	33 (54.1%)	25 (50.0%)	8 (72.8%)	
Indication for index surgery				
Cervical Spondylosis (%)	37 (60.6%)	33 (66.0%)	4 (36.4%)	0.001
SCI/Trauma (%)	5 (8.2%)	4 (8.0%)	1 (9.1%)	
Cervical Myelopathy (%)	10 (16.4%)	10 (20.0%)	0 (0.0%)	
Cervical Radiculopathy (%)	9 (14.8%)	3 (6.0%)	6 (54.5%)	
Levels in index surgery				
One level (%)	29 (47.5%)	18 (36.0%)	11 (100.0%)	0.001
Two levels (%)	27 (44.3%)	27 (54.0%)	0 (0.0%)	
Three levels (%)	5 (8.2%)	5 (10.0%)	0 (0.0%)	
Mean number of levels in index surgery ± SD	1.6 ± 0.6	1.7 ± 0.6	1.0 ± 0.0	0.001
Mean time from index surgery to revision surgery ± SD (years)	9.5 ± 6.2	9.7 ± 6.2	8.3 ± 6.3	0.001
Indication for revision surgery				
Adjacent segment disease (%)	52 (85.2%)	44 (88.0%)	8 (72.7%)	0.165
Cervical myeloradiculopathy (%)	7 (11.5%)	4 (8.0%)	3 (27.3%)	
Pseudarthrosis (%)	2 (3.3%)	2 (4.0%)	0 (0.0%)	
Mean number of levels in revision surgery ± SD	1.4 ± 0.5	1.3 ± 0.5	2.0 ± 0.5	<0.001
Level of revision surgery				
One level (%)	36 (59.0%)	35 (70.0%)	1 (9.1%)	<0.001
Two levels (%)	22 (36.1%)	14 (28.0%)	8 (72.7%)	
Three levels (%)	3 (4.9%)	1 (2.0%)	2 (18.2%)	
Level of revision surgery				
Proximal to index level (%)	31 (50.8%)	28 (56.0%)	3 (27.3%)	0.018
Distal to index level (%)	17 (27.9%)	14 (28.0%)	3 (27.3%)	
Combined (%)	10 (16.4%)	5 (10.0%)	5 (45.4%)	

There were no significant statistical differences in operative time (P = 0.868) and length of hospital stay (P = 0.234) between the two groups. The amount of blood loss was approximately 27 ml less in the ACDF-CP group than in the ACDF-ZP group without a significant statistical difference (P = 0.486) (Table 2).

**Table 2:** Comparison of patient outcomes and complications between groups *A P-value of <0.05 was considered statistically significant. PEEK, polyetheretherketone

	All patients N =61	Zero profile implant (ACDF-ZP Group) N=50 patients	Traditional PEEK interbody cage, plate, and screws (ACDF-CP Group) N=11 patients	P-value
Mean operative time ± SD (min)	122 ± 35.5	121.7 ± 35.3	124.1 ± 39.8	0.868
Mean blood loss ± SD (ml)	105.8 ± 87.1	110.2 ± 91.1	83.3 ± 51.6	0.486
Mean length of hospital stay ± SD (days)	3.0 ± 1.6	3.1 ± 1.7	2.3 ± 0.5	0.234
Intraoperative complications (%)	0 (0.0%)	0 (0.0%)	0 (0.0%)	-
In-hospital complications (%)	2 (3.3%)	2 (4.0%)	0 (0.0%)	0.793
Dysphonia (%)	8 (13.1%)	8 (16.0%)	0 (0.0%)	0.302
Surgical site infections (%)	2 (3.3%)	2 (4.0%)	0 (0.0%)	0.755
Reoperation rate (%)	3 (4.9%)	3 (6.0%)	0 (0.0%)	0.648
Mean time for the reoperation ± SD (days)	110.6 ± 65.3	110.6 ± 65.3	0	-
Follow-up complications (%)	1 (1.6%)	1 (2.0%)	0 (0.0%)	0.821

Overall, there were no significant differences in complications and reoperation rates between the two groups. There were no intraoperative complications; however, the ACDF-ZP group had two in-hospital complications (one hematoma and one seroma) and two surgical site infections which did not require any intervention. All eight cases of postoperative dysphonia occurred in the ACDF-ZP group without any long-term sequelae. Additionally, all three reoperations occurred in the ACDF-ZP group due to implant failure, one of which was associated with trauma. The mean + SD time for reoperation was 110.6 + 65.3 days. (Table 2)

The overall incidence of dysphagia decreased from 36 patients (59.0%) as an inpatient, 24 patients (39.3%) at two weeks postoperatively to 18 patients (29.5%) at six weeks postoperatively, and two patients (3.3%) after six months or more postoperatively. The in-hospital incidence of dysphagia was significantly less in the ACDF-CP group (P = 0.041). In the ACDF-ZP group, 31 patients (62.0%) reported dysphagia as an in-patient with half resolved by six weeks, two patients persisted for more than six months, and one patient required a temporary percutaneous endoscopic gastrostomy (PEG) tube. Conversely, in the ACDF-CP group, only five patients (45.5%) reported dysphagia as an inpatient with all resolved by six months postoperatively (Table 3). Overall, a majority of cases of dysphagia were transient and required no treatment (29 patients; 47.5%); whereas seven patients (11.5%) required a short course of oral steroids, and one patient required a temporary PEG tube placement.

**Table 3:** Comparison of dysphagia diagnosed in patients between groups *A P-value of <0.05 was considered statistically significant. PEEK, polyetheretherketone; PEG, percutaneous endoscopic gastrostomy

	N (%)	Zero profile implant (ACDF-ZP Group)	Traditional PEEK interbody cage, plate, and screws (ACDF-CP Group)	P-value
Number of patients (%)	61 (100.0%)	50 (82.0%)	11 (18.0%)	
In-hospital incidence (%)	36 (59.0%)	31 (62.0%)	5 (45.5%)	0.041
Incidence at two weeks follow-up (%)	24 (39.3%)	18 (36.0%)	6 (54.5%)	0.211
Incidence at six weeks follow-up (%)	18 (29.5%)	14 (28.0%)	4 (36.4%)	0.414
Incidence at more than six months follow-up (%)	2 (3.3%)	2 (4.0%)	0 (0.0%)	0.669
Treatment				
Nothing/Cepacol (%)	29(47.5%)	24 (48.0%)	5 (45.5%)	0.495
Steroids (%)	7 (11.5%)	6 (12.0%)	0 (0.0%)	
PEG tube (%)	1 (1.6%)	1 (2.0%)	0 (0.0%)	

Table 4 depicts a comparison of patient radiological parameters. Global cervical alignment improved in all included subjects regardless of the implant selection or the number of operated levels. Although more correction was observed in the ACDF-CP than in the ACDF-ZP, this was not statistically significant. At the final follow-up, the ACDF-ZP group maintained better correction than the ACDF-CP group, although the difference was not statistically significant. Regarding local alignment, there was no statistically significant difference in the correction or the maintenance of the correction. The incidence of cage subsidence was approximately 4% in the ACDF-ZP group, and there was no statistically significant difference between the two groups (P = 0.630) (Table 4).

**Table 4:** Comparison of patient radiological parameters between groups *A P-value of <0.05 was considered statistically significant. PEEK, polyetheretherketone

	All Patients N =61	Zero profile implant (ACDF-ZP Group) N=50	Traditional PEEK interbody cage, plate, and screws (ACDF-CP Group) N=11	P-value
Mean global alignment ± SD (deg)				
Preoperative	12.9 ± 8.2	14.1 ± 8.0	7.1 ± 6.4	0.017
Postoperative	15.9 ± 9.7	16.4 ± 10.2	13.5 ± 6.7	0.371
Final follow-up	15.1 ± 7.7	16.1 ± 7.9	11.2 ± 6.0	0.760
Mean local alignment ± SD (deg)				
Preoperative	8.3 ± 6.0	8.4 ± 6.2	8.0 ± 5.5	0.853
Postoperative	8.6 ± 6.2	8.5 ± 6.1	9.0 ± 6.8	0.800
Final follow-up	9.4 ± 6.2	9.6 ± 6.1	8.9 ± 6.9	0.765
Cage subsidence (% of patients)	2 (3.3%)	2 (4.0%)	0 (0.0%)	0.630

A subgroup analysis was performed for patients with adjacent segment failure, the most common indication for revision. There were 44 (88.0%) patients in the ACDF-ZP and 8 (72.7%) in the ACDF-CP group. The mean deviation (MD) for blood loss was -19.87 (95% CI: -77.68, 37.94, P = 0.50). The MD for operative time was 7.35 (95% CI: -14.47, 29.17, P = 0.51). The odds ratio (OR) of dysphagia in the ACDF-CP group compared to the ACDF-ZP group was 0.95 (95% CI: 0.20, 4.52, P = 0.95). A trial of propensity-matched analysis was carried out using age, sex, and the indication for revision, and no match was found.

## Discussion

Anterior cervical decompression and fusion procedures are generally considered safe, although it is important to note that a wide range of complications are possible, including ASD, which is associated with significant rates of revision [6,8]. Hilibrand et al. found the survivorship analysis predicted that 25.6% of patients would develop ASD within ten years of anterior cervical arthrodesis, with greater than two-thirds of these patients requiring revision surgeries [11]. In our study, ASD was a factor for the majority (85.2%) of revision surgeries.

The stand-alone implant was designed to reduce complications associated with anterior cervical fusion [7]. Stand-alone implants may provide additional benefits when utilized in revision surgeries. Revision surgeries can result in additional violations of soft tissue and adjacent levels when removing hardware from the index surgery [12]. A few studies have compared the stand-alone implant and traditional instrumentation options for revision ACDF [10, 12-14]. Our findings indicate outcomes and complications of revision ACDF are similar when utilizing a stand-alone implant or a traditional interbody PEEK cage, titanium plate, and screw instrumentation.

Postoperative dysphagia is the most common complication following ACDF procedures, which is thought to be multifactorial, with dislodged graft, hardware protrusion, nerve injury, soft tissue swelling, hematoma formation, and esophageal edema as potential contributing factors [10,15,16]. Risk factors for developing postoperative dysphagia include patient age, operative time of the procedure, female, smoking, and baseline dysphagia [17-19]. Patients who develop postoperative dysphagia after a primary operation may be more likely to develop this phenomenon after revision surgery [14]. Further, when utilizing traditional interbody cage, plate, and screws for revision, the anterior plate from the initial surgery is often removed, potentially contributing to higher rates of dysphagia [14]. Reported rates of postoperative dysphagia range from 5% to 71% [15-20]. Stand-alone implants have demonstrated decreased incidence in primary ACDF surgeries [7]. Few studies have demonstrated similar findings for revision ACDF for ASD [10,13]. Niu et al. reported a statistically significant difference in postoperative dysphagia with 57.69% in the traditional cage and plate construct group and 18.75% in the stand-alone group following revision ACDF [14].

Overall, postoperative dysphagia was experienced by 36 (59.0%) patients in our study. In contrast to previous studies, our data demonstrated a greater occurrence of postoperative dysphagia in the ACDF-ZP group (62.0%) compared to 45.5% in the ACDF-CP group. This finding may be explained in part due to the small sample size of the ACDF-CP group and the subjective nature of dysphagia. Additionally, these procedures were completed by two different surgeons with slightly varying techniques which may contribute to our findings. Lastly, the variance may also be explained by established pre-existing patient-specific risk factors for postoperative dysphagia such as the number of levels to be treated, gender, cephalad levels, and history of dysphagia, among others which were not accounted for in this study.

Edwards et al. found surgeons underreported dysphagia in patient charts when compared to patient surveys following anterior cervical arthrodesis procedures [21]. Any study assessing postoperative dysphagia may be influenced by underreporting. The rates of overall dysphagia declined by the two- and six-weeks follow-up. At these time points, dysphagia was greater in the ACDF-CP group, although the difference was not statistically significant. Only two patients’ dysphagia persisted for greater than six months, both in the ACDF-ZP group. The declining rates of dysphagia in the follow-up period align with previous research demonstrating that postoperative dysphagia is transient with a low incidence at long-term follow-up [13,14,18,19]. The low rates of long-term dysphagia in the ACDF-CP and ACDF-ZP groups suggest that both options are acceptable for revision ACDF.

Due to the additional challenges of revision surgery, a shorter operative time with less interference is desired [14]. Our study reported no significant differences in blood loss or operative time, indicating that both instrumentation options are reasonable selections for revision. Our results are similar to Gandhi et al. who found no differences in operative time or estimated blood loss between the anterior plate construct and stand-alone groups for revision ACDF for ASD [12]. Contrary to these results, other studies comparing these instrumentation options have reported shorter operative times or decreased blood loss with stand-alone implants in both primary and revision surgeries [7,10,13,14]. In our study, there was no difference in the length of hospital stay between groups, similar to the findings reported by Niu et al. [14].

Cervical sagittal balance is an important postoperative clinical measure, as loss of cervical lordosis can cause pain and dysfunction, and negatively impact global spinal alignment [22,23]. In this study, local and global alignments were utilized to assess postoperative cervical sagittal balance, defined as the Cobb angle of the operated level and the C2-C7 Cobb angle, respectively. Both the ACDF-CP and ACDF-ZP groups demonstrated improvement postoperatively and at final follow-up, with no statistically significant difference between groups. These findings are similar to other studies that found improvement in the C2-C7 angle utilizing stand-alone implants and traditional instrumentation options [13,14]. Cage subsidence is another crucial consideration when utilizing stand-alone implants, which can lead to sagittal imbalance [14,24]. Two patients in the ACDF-ZP group from our study experienced cage subsidence. However, there was no statistically significant difference between the ACDF-ZP and ACDF-CP groups. Cage subsidence was observed only in the ACDF-ZP group, presumably due to the considerable difference in sample sizes between groups. Shen et al. also reported no statistically significant difference in cage subsidence between zero-profile implants and traditional options for revision ACDF [13]. Our results from the tested radiological parameters indicate there was no difference in correction between stand-alone implants and traditional interbody cage, plate, and screw options for revision ACDF with comparably low rates of cage subsidence.

Overall, there were no differences in the rates of complications between groups. All complications and reoperations occurred in the ACDF-ZP group, likely due to the larger sample size of the group. Two complications occurred in the hospital postoperatively, a hematoma and a seroma. Further, the ACDF-ZP group included eight patients who developed transient dysphonia, two with surgical site infections, and three reoperations for hardware failure. Gandhi et al. found no difference in reoperation rates between stand-alone cages and anterior plate constructs for revision ACDF [12]. However, the authors found that the stand-alone cage had a significantly lower rate of fusion and higher rates of reoperation in revision ACDF when compared to primary ACDF patients. 

Our study has several limitations. Only 11 patients with revision surgery utilized a traditional interbody cage, plate, and screws resulting in a relatively small sample size for the ACDF-CP group compared to the ACDF-ZP group. This is likely explained by the fact that the surgeon who utilizes ACDF-CP for revision has a greater tendency to perform revision cervical procedures posteriorly versus anterior revision approaches. Additionally, long-term follow-up was not achieved in all patients in this study, as postoperative dysphagia was the primary measure. Further, procedures in each group were performed by surgeons with slightly varying techniques. In addition, established pre-existing risk factors were not stratified in this study. As this study was conducted retrospectively, no preoperative dysphagia assessments were obtained. Finally, our study also carries the risk of potential limitations inherent to retrospective studies.

The strength of this study was the large sample size, specifically the considerable sample size of the stand-alone implant group compared to previous studies. Further, comprehensive data collection allowed for in-depth intraoperative, postoperative, and radiographic comparisons. Finally, patient characteristics, demographics, and indications for revision procedures were similar between groups, resulting in a reliable comparison of these instrumentation options for revision in the setting of adjacent segment failure.

## Conclusions

Rates of dysphagia were comparable between groups at short and long-term follow-up, despite a greater incidence of postoperative dysphagia in the ACDF-ZP group. All complications and occurrences of cage subsidence were observed in the ACDF-ZP group. This may be attributed to the larger sample size of patients within this group. Given our findings, zero-profile stand-alone implants and traditional interbody PEEK cage, titanium plate, and screw instrumentation appear to be both safe and effective options for revision ACDF.
